# Artificial intelligence for HIV care: a global systematic review of current studies and emerging trends

**DOI:** 10.1002/jia2.70045

**Published:** 2025-09-24

**Authors:** Sanele Ngcobo, Edith Madela Mntla, Jonathan Shock, Murray Louw, Linda Mbonambi, Thato Serite, Theresa Rossouw

**Affiliations:** ^1^ Department of Family Medicine University of Pretoria Pretoria South Africa; ^2^ Department of Mathematics and Applied Mathematics University of Cape Town Cape Town South Africa; ^3^ INRS Montreal Quebec Canada; ^4^ NITheCS Stellenbosch South Africa; ^5^ Library University of Pretoria Pretoria South Africa; ^6^ Department of Immunology University of Pretoria Pretoria South Africa

**Keywords:** artificial intelligence, chatbots, diagnostics, HIV care, machine learning, retention to care, treatment monitoring, viral suppression

## Abstract

**Introduction:**

Artificial intelligence (AI) and, in particular, machine learning (ML) have emerged as transformative tools in HIV care, driving advancements in diagnostics, treatment monitoring and patient management. The present review aimed to systematically identify, map and synthesize studies on the use of AI methods across the HIV care continuum, including applications in HIV testing and linkage to care, treatment monitoring, retention in care, and management of clinical and immunological outcomes.

**Methods:**

A comprehensive literature search was conducted across databases, including PubMed and ProQuest Central, Scopus and Web of Science, covering studies published between 2014 and 2024. The review followed PRISMA guidelines, screening 3185 records, of which 47 studies were included in the final analysis.

**Results:**

Forty‐seven studies were grouped into four thematic areas: (1) HIV testing, AI models improved diagnostic accuracy, with ML achieving up to 100% sensitivity and 98.8% specificity in self‐testing and outperforming human interpretation of rapid tests; (2) Retention in care and virological response, ML predicted clinic attendance, viral suppression and virological failure (72–97% accuracy; area under the curve up to 0.76), enabling early identification of high‐risk patients; (3) Clinical and immunological outcomes, AI predicted disease progression, immune recovery, comorbidities and HIV complications, achieving up to 97% CD4 status accuracy and outperforming clinicians in tuberculosis diagnosis; (4) Testing and treatment support, AI chatbots improved self‐testing uptake, linkage to care and adherence support. Methods included random forests, neural networks, support vector machines, deep learning and many others.

**Discussion:**

AI has the potential to transform HIV care by improving early diagnosis, treatment adherence and retention in care. However, challenges such as data quality, infrastructure limitations and ethical considerations must be addressed to ensure successful implementation.

**Conclusions:**

AI has demonstrated immense potential to address gaps in HIV care, improving diagnostic accuracy, enhancing retention strategies and supporting effective treatment monitoring. These advancements contribute towards achieving the UNAIDS 95‐95‐95 targets. However, challenges such as data quality and integration into healthcare systems remain. Future research should prioritize scalable AI solutions tailored to high‐burden, resource‐limited settings to maximize their impact on global HIV care.

**PROSPERO Number:**

PROSPERO 2024 CRD42024517798 Available from: https://www.crd.york.ac.uk/prospero/display_record.php?ID=CRD42024517798

## 1 INTRODUCTION

The HIV pandemic has had devastating consequences. Since its onset, an estimated 85.6 million people have acquired HIV, and 40.4 million people have died from AIDS‐related causes. In 2022, approximately 39 million people globally were living with HIV. In the same year, approximately 1.3 million individuals newly acquired HIV, while 630,000 lost their lives to AIDS‐related illnesses [1]. Notably, nearly 29.8 million people had access to antiretroviral therapy (ART) [1], and UNAIDS estimates revealed considerable progress towards the 95‐95‐95 targets. The achievement of 86%‐89%‐93% (86% of people living with HIV [PLWH] knowing their status, 89% of those with a known status on treatment and 93% of those on treatment achieving viral suppression) is a significant improvement from the 71%‐67%‐83% estimates of 2015 [1]. Much work, however, still needs to be done: at the end of 2023, ±5.5 million PLWH were not yet aware of their status; ±9 million were not on ART; and ±11.3 million were not virally suppressed. To attain the global HIV targets, innovative strategies will be needed.

Artificial intelligence (AI) is at the forefront of global innovation, with its potential to enhance health outcomes gaining increasing recognition. AI refers to the capacity of machines, such as computers, to replicate aspects of human intelligence [2]. Machine learning (ML), a subset of AI, entails the utilization of algorithms and statistical models programmed to learn from data, thereby discerning patterns and executing tasks without explicit human instructions [2]. Supervised learning, a type of ML, learns to map inputs to outputs through pre‐matched (labelled) pairs, contrasting with unsupervised learning, which identifies patterns in data without prior labelling [2]. Neural networks, inspired by biological systems, employ interconnected neurons with adjustable weights to make predictions, while deep learning refers to neural networks with multiple hidden layers of neurons [2]. These concepts collectively underpin the potential of AI to revolutionize various fields, including healthcare, by enhancing decision‐making processes and ultimately improving outcomes [2].

AI's integration into clinical HIV care offers promise for the improvement of disease diagnosis, treatment selection and risk assessment. This has the potential to enable healthcare systems to more accurately identify PLWH, initiate ART in a timely manner and implement targeted retention strategies, ultimately improving patient and public health outcomes. While systematic reviews have been conducted on the role of AI in managing conditions like hypertension and diabetes, there does not appear to have been an exploration of the potential applications of AI in various aspects of HIV care [3, 4, 5, 6]. This study aimed to systematically identify, map and synthesize studies on the use of AI methods across the HIV care continuum, including applications in HIV testing and linkage to care, treatment monitoring, retention in care, and management of clinical and immunological outcomes.

## 2 METHODS

This systematic review followed the Preferred Reporting Items for Systematic reviews and Meta‐Analyses (PRISMA) guidelines [7]. The systematic review was registered with PROSPERO to ensure transparency and adherence to the predefined methodology and reporting standards (https://www.crd.york.ac.uk/PROSPERO/display_record.php?RecordID=517798).

### 2.1 Search strategy

The search strategy was developed by a librarian, author LM, in consultation with SN. This search strategy was designed to gather relevant articles on HIV care and the application of AI in healthcare, spanning the period from 2014 to 2024. It included queries in four key databases: PubMed, Scopus, Web of Science and ProQuest Central. The search terms focused on HIV‐related care and treatment, including HIV management, ART and HIV/AIDS interventions, paired with terms related to AI and healthcare technologies such as ML, automated systems and digital health. Filters for article type, language (English) and time span (2014−2024) were applied to ensure the results were up to date and relevant. The search results from these databases included 265 articles from PubMed, 355 from Scopus, 314 from Web of Science and 2799 from ProQuest Central. The detailed search strategy is provided in Supplementary Material 1.

### 2.2 Screening

**Figure 1:**
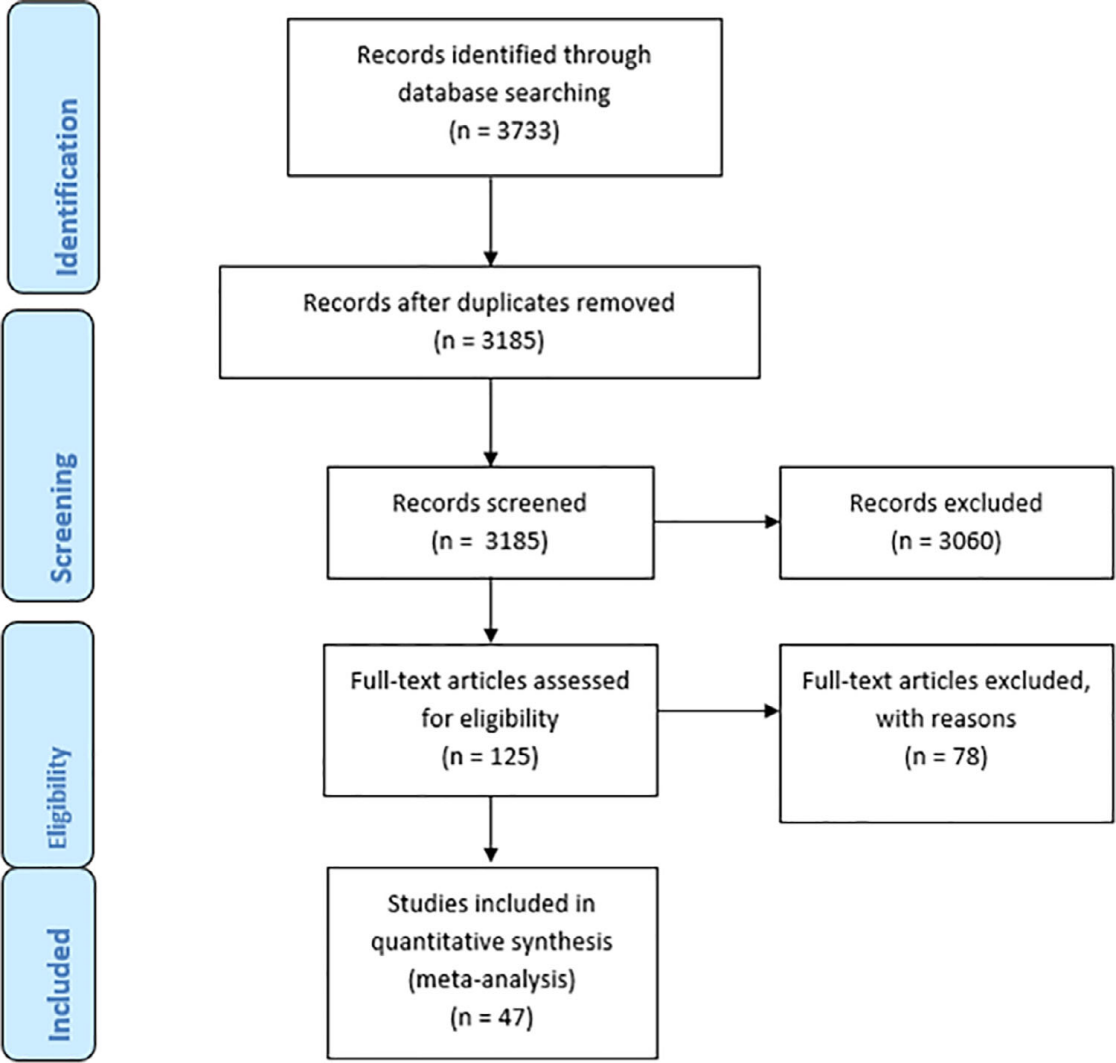
**PRISMA 2020 flow diagram of study selection**.

In accordance with PRISMA guidelines, a total of 3733 records were identified through database searching. After duplicates were removed, 3185 records remained for screening eligibility. The title and abstract screening phase was conducted by two authors, SN and TB, who reviewed the 3185 remaining records based on predefined inclusion and exclusion criteria. Of these, 3060 records were excluded based on predefined criteria. Subsequently, 125 full‐text articles were assessed for eligibility, of which 74 were excluded for various reasons such as irrelevant study designs, populations that did not focus on PLWH or interventions that were not related to AI in HIV care. Ultimately, 47 studies were included in the narrative synthesis for analysis (Figure 1).

### 2.3 Study selection criteria

This systematic review included observational studies, experimental trials and studies that investigated AI applications in HIV care. We defined HIV care to encompass clinical activities and outcomes following HIV diagnosis, including ART initiation, adherence and retention, viral suppression, immunologic monitoring (e.g. CD4/CD8 ratios), management of comorbidities, prediction of treatment‐related outcomes (e.g. frailty, mortality, coinfections) and clinical decision support for ongoing care. While our search strategy primarily targeted HIV care and management terms, we also included studies on AI interventions that facilitated entry into care through mechanisms such as HIV self‐testing promotion that was linked to clinical follow‐up or ongoing care engagement (e.g. AI chatbots promoting self‐testing with linkage to services). The inclusion criteria were established to capture a comprehensive range of AI applications in HIV diagnosis, treatment monitoring and patient management. Studies were excluded if they were published in languages other than English, were conducted before 2014 or focused on AI applications without a direct connection to HIV care. Additionally, studies that applied AI techniques but did not report outcomes related to HIV care were excluded to ensure relevance.

### 2.4 Data collection process

The data extraction process followed a structured approach to maintain accuracy and consistency. SN and TS independently extracted data, and the compiled dataset was subsequently reviewed and refined by author ML, who acted as the third reviewer. Given the complexity of ML methodologies, JS, a mathematician specializing in AI, conducted an independent review of the extracted ML techniques to ensure precise classification and correct interpretation of algorithmic approaches. To enhance consistency, the research team conducted virtual consensus meetings, where reviewers discussed discrepancies and reached a final agreement on study inclusion and data categorization. Data extraction adhered to PRISMA guidelines and was systematically recorded in Microsoft Excel. The extracted variables included: Study ID (Article Title), File Name, ML Techniques Used, Study Design, Study Setting, Study Population/Participants, Sample Size, Intervention/Exposure, Comparator/Control, Outcomes of Interest, Effect Size/Measure of Association, Statistical Methods, Key Findings/Results and Conclusion/Summary. This structured approach facilitated a comprehensive synthesis of the data while ensuring consistency across studies. ML techniques were categorized using standard taxonomies found in authoritative sources such as Scikit‐learn, The Elements of Statistical Learning and Topol's framework for AI in healthcare.

### 2.5 Quality appraisal

The methodological rigour of the included studies was assessed using the Critical Appraisal Skills Programme (CASP) checklist (Supplementary Material 2) [8]. TS evaluated the studies, focusing on key aspects such as study design, risk of bias, validity and generalizability. Any discrepancies in quality assessment were discussed and resolved through consensus to maintain the integrity of the review.

### 2.6 Synthesis strategy

The synthesis of findings was structured around four key thematic areas that reflected the different applications of AI in HIV care. Studies were categorized into: (1) HIV testing; (2) Retention in care and virological treatment response; (3) Clinical and immunological treatment outcomes; and (4) Testing and treatment support. In parallel, ML techniques were analysed based on the ML methods employed, such as random forests (RFs), logistic regression (LR), support vector machines (SVMs) and neural networks, to map their usage in various aspects of HIV care.

## 3 RESULTS

**Figure 2:**
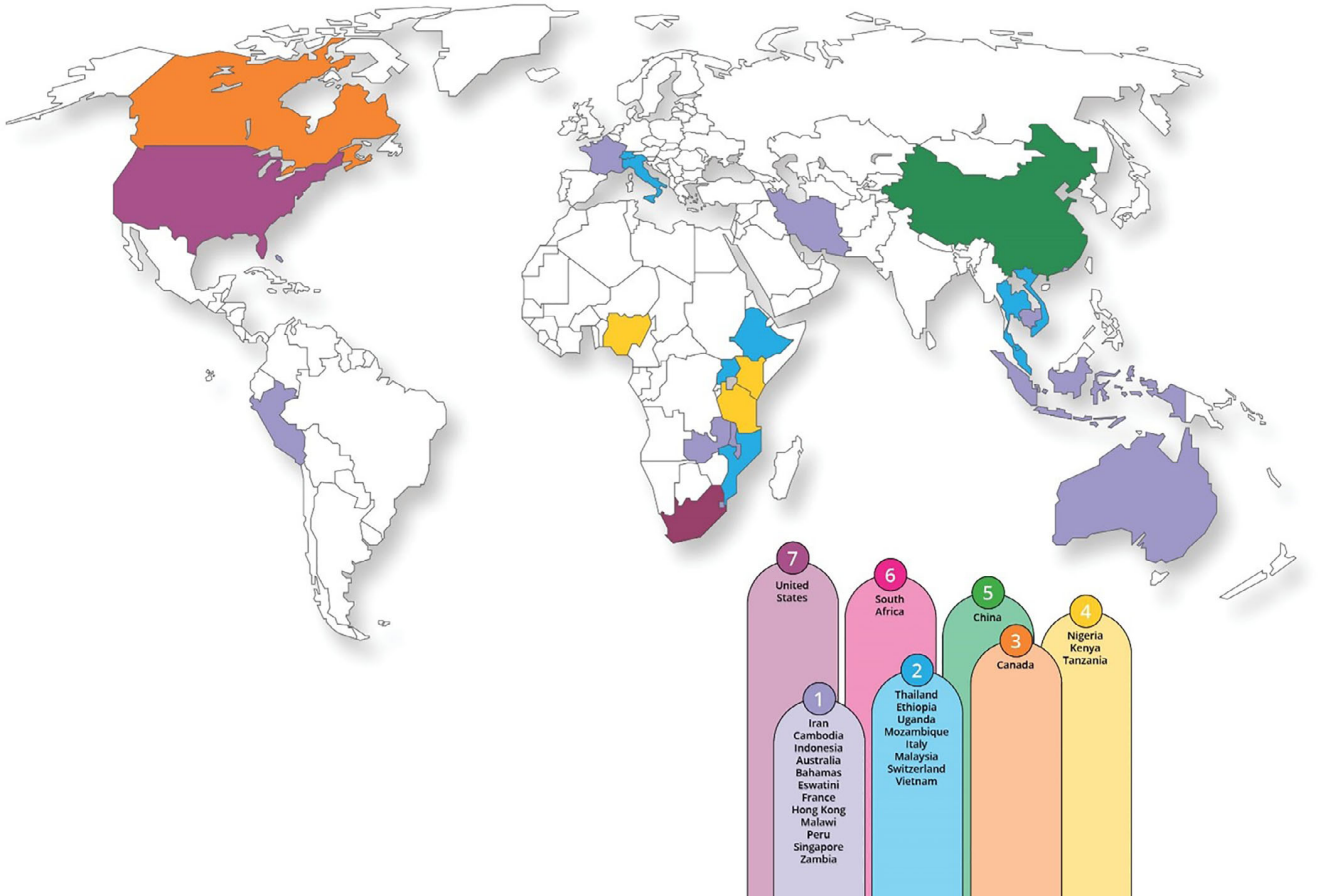
**Geographic coverage of included studies on AI in HIV care**.

Table 1 summarizes the 47 included studies, detailing authors, study design, setting, population, sample size, outcomes and AI methods used. Figure 2 presents the country distribution of included studies, with the highest representation from the United States (*n* = 7), South Africa (*n* = 6), China (*n* = 5), Canada (*n* = 3), and Kenya, Nigeria and Tanzania (*n* = 4). Studies were conducted across both high‐ and low‐ to middle‐income countries, reflecting the global interest in applying AI methods across the HIV care continuum. Study designs ranged from retrospective and prospective cohorts to randomized controlled trials and cross‐sectional analyses.*

**Table 1:** Details of the studies included in the review

Ref	Author	Study design	Country/region	Population	Sample size^a^	AI methods used	Key findings
[9]	Jeni et al. (2022)	Retrospective study	Nigeria and Mozambique	PLWH	360	Logistic regression, ANN, random forest, XGBoost, extra trees	Random forest, boosted trees predicted ART loss to follow‐up with AUPRC = 0.65 in Mozambique and 0.52 in Nigeria, outperforming standard methods and supporting targeted interventions across sex and age groups.
[10]	Tu et al. (2021)	Longitudinal study	Canada	PLWH: three patient groups: peripheral neuropathy (*n* = 111)—included HIV‐associated distal sensory polyneuropathy (*n* = 90); mononeuropathy (*n* = 21); and non‐neuropathy (*n* = 408)	540	Random forest, logistic regression	Random forest models (AUC > 0.80) outperformed logistic regression in predicting peripheral neuropathy, with HIV‐1 duration, peak viral load, age and low CD4+ T‐cell count as top predictors.
[11]	Roy et al. (2022)	Cohort study	Canada	PLWH	186	Random forest, logistic regression	Random forest models (AUC = 0.76–0.79) outperformed logistic regression (*p* = 0.0001) in predicting carotid plaques using age, smoking, axial strain, pulse pressure and HIV status.
[12]	Domínguez‐Rodríguez et al. (2022)	Prospective study	South Africa and Mozambique	Children with perinatally acquired HIV who began ART <3 months of age	100	Random forest, SVM, k‐nearest neighbours, naïve Bayes, ANN, elastic net, logistic regression	Random forest achieved 82.8% accuracy, 78% sensitivity and AUC = 0.73 in predicting 1‐year mortality or AIDS progression, outperforming six other models including logistic regression.
[13]	Federico et al. (2023)	Observational study	Italy	PLWH	3321	Gradient boosting, SHAP (explainability)	Gradient boosting, random forest and support vector regression predicted >5% weight gain with >90% accuracy when body composition data (DEXA) were included; simplified models were sufficient to rule out gainers.
[14]	Esber et al. (2023)	Cohort study	Sub‐Saharan Africa: Uganda, Kenya, Tanzania and Nigeria	PLWH	2941	LASSO regression, random forest	LASSO regression (AUC = 0.82) and random forest (AUC = 0.75) predicted viral failure using 94 variables; key predictors included CD4 count, ART regimen, adherence, age and duration on ART.
[15]	Fahey et al. (2022)	Cohort study	Tanzania	PLWH	178	Ensemble decision trees	An ensemble decision tree model using routine EMR data predicted 6‐month disengagement from care (≥28‐day no‐show) with 75.2% accuracy and 54.7% sensitivity among the top 30% highest risk group.
[16]	Kagendi and Mwau (2023)	Cross‐sectional study	Kenya	Pseudonymized participants	4 million tests and 4265 facilities	Random forest	Using 4 million VL records from 4265 Kenyan health facilities, a random forest model predicted viral load hotspots (≥20% unsuppressed) with 78% accuracy, F1 score = 69% and Brier score = 0.139, correctly identifying 434 of 446 hotspots.
[17]	Mamo et al. (2023)	Cross‐sectional study	Ethiopia	PLWH	5264	Random forest, association rule mining	A random forest model predicted virological failure with AUC = 0.9989, sensitivity = 1.00 and F1‐score = 0.993; top predictors included low CD4 count, ART duration, TDF‐3TC‐EFV regimen and lack of CPT/TPT use.
[18]	Maskew et al. (2022)	Longitudinal study	South Africa	PLWH	445,636 patients (retention model) 363,977 (viral load model)	Logistic regression, random forest, AdaBoost	Random forest, logistic regression and AdaBoost predicted next‐visit attendance (AUC = 0.69) and viral load suppression (AUC = 0.76), with key predictors including prior late visits, treatment duration and number of prior VL tests.
[19]	Poorinmohammad and Mohabatkar (2015)	Descriptive study	Iran	Three datasets: positive dataset, experimentally tested peptides and independent test sets	230, 212 and 47	ANN, logistic model tree, random forest, K star, J48 decision tree	Using pseudo amino acid composition features, MLP and logistic model tree algorithms predicted anti‐HIV‐1 peptides with 96.15% and 83.71% accuracy, respectively, making MLP the most accurate classifier for sequence‐based peptide prediction.
[20]	Seboka et al. (2023)	Retrospective study	Ethiopia	PLWH	2907	K‐nearest neighbours (KNN) Support vector machine (SVM) Logistic regression (LR) Decision tree (DT) Gaussian naive Bayes (GNB) Random forest (RF) Gradient boosting (GB) eXtreme gradient boosting (XGB)	XGB predicted viral load >1000 copies/ml with 96% accuracy, 97% sensitivity and AUC = 0.99, while GB achieved the highest accuracy for CD4 <200 classification; RF ranked second in viral load prediction.
[21]	Steiner et al. (2020)	Retrospective study	USA	HIV‐1 sequence data and drug resistance assay	No study population. 18 ART drugs	Multilayer perceptron (MLP), bidirectional recurrent neural network (BRNN), convolutional neural network (CNN)	CNN achieved the highest drug resistance classification accuracy (mean AUC = 0.95), outperforming MLP (AUC = 0.91) and BRNNs (AUC = 0.93), with model interpretability confirming importance of key drug resistance mutations.
[22]	Xianglong et al. (2022)	Retrospective study	Australia	Men who have sex with men	1627	Logistic regression, LASSO, ridge regression, elastic net, SVM, k‐nearest neighbour, naïve Bayes, random forest, XGBoost, MLP	XGBoost predicted timely post‐reminder clinic attendance (AUC = 62.8%, F1 = 70.8%), while elastic net regression best predicted HIV/STI testing uptake within 30 days (AUC = 82.7%, F1 = 85.3%).
[23]	Peng et al. (2022)	Qualitative study	Malaysia	Men who have sex with men in Malaysia	Five web‐based focus group interviews: 31 MSM	NLP	Participants expressed interest in AI chatbots for HIV prevention if anonymity, accurate information, linkage to services and stigma‐sensitive design were ensured; concerns included privacy, usability and legal risks.
[24]	Murnane et al. (2021)	Retrospective study	Sub‐Saharan Africa	Pregnant women (2011–2014)	1321	LASSO, logistic regression, SuperLearner, random forests, gradient boosting	SuperLearner and LASSO models using routine clinical data predicted viraemia (>50–1000 copies/ml) with moderate accuracy (AUC = 0.74–0.78), but flagged 64% of women as high risk to achieve 90% sensitivity—limiting utility for targeted interventions.
[25]	MacPherson et al. (2021)	Randomized controlled trial	Malawi	Adults (≥18 years) with cough attending acute primary services in Malawi	473, 492 and 497	ANN	HIV‐TB screening with computer‐aided chest X‐ray (CAD4TBv5) plus universal HIV testing led to faster TB treatment initiation (median = 1 vs. 11 days in SOC) and 40% same‐day TB treatment versus 0% in SOC; undiagnosed HIV dropped from 2.7% to 0.2% (RR = 0.09).
[26]	Koh et al. (2024)	Qualitative evaluation of chatbot responses	Singapore	Simulated patient queries	23 prompts	NLP	ChatGPT provided accurate and comprehensive responses to ART‐related queries, demonstrating utility as a supportive tool for HIV counselling, though its advice remained generic and required clinical contextualization.
[27]	Olatosi et al. (2021)	Retrospective study	USA	PLWH	8888	Bayesian network, random forest, decision trees, ANN	ML models (SVM, RF, MLP) predicted CD4/CD8 ratio from clinical data; SVM performed best for CD4<200 (*R* ^2^ = 0.365), RF best for CD4≥200 (*R* ^2^ = 0.341).
[28]	Semenova et al. (2023)	Cross‐sectional study	USA	PLWH	115	Linear regression, RF, PaCMAP (pairwise controlled manifold approximation projection) GOSDT (global optimal sparse decision trees), LOCO (leave‐one‐covariate‐out) Inference	RF and LR models were applied to immunologic and reservoir data from 115 ART‐treated individuals; prediction of high versus low total or intact HIV DNA yielded ∼70% balanced accuracy across models. Notably, CD127 expression on CD4+ T cells and years on ART were strong features in classifying reservoir level.
[29]	Aybar‐Flores et al. (2023)	Retrospective study	Peru	Adolescents and young people	10,565	Random forest, quasi‐binomial logistic regression, k‐nearest neighbours, decision tree, artificial neural network	Random forest performed best (accuracy = 64.3%) to predict HIV/AIDS knowledge, identifying 14 significant predictors: gender, area of residence, wealth index, region of residence, age, highest educational level, ethnic self‐perception, having heard about HIV/AIDS, HIV/AIDS screening test history, mass media access, marital status, working status, health insurance coverage and internet use.
[30]	Bala et al. (2023)	Retrospective study	Nigeria	PLWH	2500	ANN, ANFIS, SVM, multiple linear regression (MLR)	ANFIS outperformed all models in predicting ART outcomes, with *R* ^2^ = 0.903 (training), 0.904 (testing) and MSE = 7.961 (training), 3.751 (testing); CA showed the strongest association between ART drugs and marital status (explaining 93.7% of variation).
[31]	Bisaso et al. (2018)	Observational study	Uganda	PLWH	EFV cohort = 233; IDI cohort = 484	Multitask temporal logistic regression (MTLR), patient‐specific survival prediction (PSSP), simple logistic regression (SLR)	MTLR outperformed PSSP and SLR in predicting early virological suppression (AUROC = 0.92 vs. 0.75 vs. 0.53; Brier = 0.08 vs. 0.19 vs. 0.11); external validation showed 92.9% accuracy, AUROC = 0.878 and false positive rate = 6.9%.
[32]	Chikusi et al. (2022)	Observational study	Tanzania	HIV index clients and their notified contacts	6346 HIV index clients and 7226 contacts	Random forest, XGBoost, artificial neural network (ANN)	RF performed best (MAE = 1.1261) in predicting HIV index testing outcomes compared to XGBoost (MAE = 1.2340) and ANN (MAE = 1.1268); model visualizations identified Kilimanjaro as having the highest partner‐notified cases and 82.6% of notified contacts were female.
[33]	Cotugno et al. (2020)	Cohort study	Italy	PLWH	23	Elastic net (glmnet function in R) [20], support vector machines (svm.fs function in R) [21] and random forests	An AdaBoost model using expression profiles from 46 genes across five lymphocyte subsets and conditions predicted vaccine immunogenicity with 95.6% accuracy and only one misclassified case, demonstrating high potential to identify non‐responders to influenza vaccination among children living with HIV.
[34]	Rachel et al. (2023)	Cross‐sectional study	South Africa	Patients from public health clinics	202,817	Categorical boosting and ADABoost	The categorical boosting model achieved 62% sensitivity, 67% specificity and 20% PPV, predicting 22,119 of 35,985 treatment interruptions; performance was highest during the first 6 months of ART.
[35]	Li et al. (2022)	Retrospective study	China	PLWH	96	SVM, random forest, ANN	SVM, RF and MLP models using clinical indicators effectively predicted immune function (CD4/CD8 ratio) in long‐term ART patients; SVM outperformed in low‐CD4 (<200) cases, RF performed best in higher CD4 (≥200) cases.
[36]	Li and Li (2020)	Qualitative study	China	Morbidity models	NA	ANN, ARIMA (auto‐regressive integrated moving average model)	The BP‐ANN model achieved a mean absolute percentage error (MAPE) of 3.08%, outperforming the ARIMA model with an MAPE of 7.92%, indicating superior prediction accuracy for monthly AIDS incidence in Henan Province, China, from 2004 to 2016.
[37]	Luckett et al. (2021)	Cross‐sectional study	USA	297 PLWH and 1509 HIV‐negative controls	297	ANN	The salience (SAL), parietal memory (PMN) and frontoparietal (FPN) networks had the highest predictive weights (up to 1.0) for classifying HIV status and cognitive impairment.
[38]	Luckett et al. (2019)	Cross‐sectional study	USA	PLWH	125	ANN	Deep neural networks (DNNs) classified cognitive impairment among PLWH with 82–86% accuracy (AUC = 0.81–0.87) and frailty status with 75% accuracy, using cerebral blood flow features from key cortical and subcortical regions like the amygdala, hippocampus, thalamus, and temporal and parietal lobes.
[39]	Marathe et al. (2022)	Mixed‐methods study	Canada	PLWH with hepatitis C virus	2018 HIV‐HCV co‐infection; 717 food security sub‐study (1934 total visits)	Random forest, feature importance analysis	A random forest model predicted depressive symptoms (CES‐D‐10 ≥10) in individuals with HIV and HCV with AUC = 0.82 using 137 predictors; key predictors were employment, HIV stage, revenue source, BMI and education.
[40]	Cheah et al. (2024)	Mixed‐methods study	Malaysia	Men who have sex with men	14	NLP, local language adaptation	An AI chatbot promoting HIV testing and PrEP among Malaysian MSM was found feasible and acceptable, with 93% finding it useful and 79% willing to reuse it; users valued its stigma‐free, user‐friendly design and suggested adding local language and more health content.
[41]	Mulyadi and Qomariyah (2023)	Retrospective secondary data analysis	Indonesia	National‐level HIV surveillance data: ART coverage (%), HIV cases, HIV‐related deaths, total population per country	75 countries	K‐nearest neighbours, logistic regression, support vector machine, XGBoost, decision tree, random forest, K‐means clustering	Random forest achieved the highest accuracy (90%) in predicting ART outcomes, while K‐means identified two clusters (15 vs. 60 countries) based on ART coverage and HIV prevalence.
[42]	Mutai et al. (2021)	Retrospective study	Sub‐Saharan Africa: Tanzania, Zambia, Malawi and Eswatini	Individuals tested for HIV	263,829	Agglomerative hierarchical clustering	XGBoost predicted HIV status with 96% accuracy and AUC = 0.99; top predictors included age, number of lifetime partners, HIV testing history and condom use.
[43]	Mutai et al. (2023)	Longitudinal study	13 sub‐Saharan African countries	Population‐based HIV impact assessment data	146,733 male and 155,622 female	Agglomerative hierarchical clustering	Two country clusters (per sex) were identified using agglomerative hierarchical clustering and PCA on 302,355 participants from 13 sub‐Saharan African countries; HIV positivity varied significantly by predictors like school enrollment, urban residence and known HIV status.
[44]	Paul et al. (2020)	Randomized controlled trial	Thailand Cambodia Vietnam Indonesia	Children with perinatally acquired HIV	285	Gradient‐boosted multivariate regression	GBM models predicted neurocognitive trajectories with 79% AUC at baseline and up to 90% with longitudinal data; mental health, hematocrit and CD4 count were key predictors.
[45]	Paul et al. (2020)	Cohort study	USA	PLWH	105	Gradient‐boosted multivariate regression	GBM identified frailty with 71% F1 score, 84% precision and 66% sensitivity; key predictors included low CD4, poor psychomotor function and visuomotor neuroimaging features, with added risk from female sex and depressive symptoms.
[46]	Pranav et al. (2020)	Cross‐sectional study	South Africa	PLWH with suspected tuberculosis	677	CNN	Deep learning model achieved 79% accuracy versus 65% for clinicians assisted by the model (*p* = 0.002), showing it significantly improves TB diagnosis in PLWH patients using chest X‐rays.
[47]	Roche et al. (2024)	Observational study	Kenya	PLWH	1500	Object detection (YOLOX Nano), classification (MixNet), image quality assurance	AI algorithm interpreting HIV self‐test images achieved 100% sensitivity, 98.8% specificity, 100% NPV and 81.5% PPV, outperforming both clients (93.2% sensitivity) and providers (97.7%) in detecting true positives.
[48]	Andresen et al. (2022)	Longitudinal study	Switzerland	Men who have sex with men	6354	Hierarchical clustering, regression analysis	Unsupervised ML clustering of MSM living with HIV identified behaviour‐based subgroups that improved prediction of 10 sexual behaviour and STI outcomes (e.g. condomless sex, syphilis), with AUROC increases of 0.03–0.17 over models using individual predictors alone.
[49]	Shi et al. (2022)	Observational study	China	PLWH with talaromycosis	1927	Logistic regression, XGBoost, k‐nearest neighbours (k‐NN), SVM	XGBoost model predicted in‐hospital mortality with 0.90 AUC, 0.69 sensitivity and 0.96 specificity; key predictors included septic shock, respiratory failure, UA, urea, PLT count and AST/ALT ratio.
[50]	Turbé et al. (2021)	Feasibility study	South Africa	HIV rapid test images	11,374	CNN	A deep learning model trained on 11,374 HIV rapid test images collected in rural South Africa achieved 97.8% sensitivity and 100% specificity in field deployment, outperforming human readers and supporting REASSURED diagnostic principles for scalable, connected HIV testing.
[51]	Wang et al. (2023)	Randomized controlled trial	The Bahamas	Grade 10–12 students	2564	Support vector machine (SVM), logistic regression, decision tree, random forest, Boruta feature selection	Random forest predicted intervention non‐responsiveness with 84.3% sensitivity, 67.1% specificity, AUROC 0.85; key predictors included self‐efficacy, parent monitoring and HIV knowledge.
[52]	Wu et al. (2023)	Retrospective study	China	PLWH	498	SVM with radial basis kernel	SVM radial model using 10 clinical factors (e.g. low Hb, CD4+, PLT; high LDH, BUN; no ART; imaging findings) predicted BSI in PLWH with high performance (AUC = 0.916, sensitivity = 82.4%, specificity = 85.5%).
[53]	Yang et al. (2021)	Cohort study	USA	PLWH	8253	LASSO regression	A LASSO regression model predicted increased comorbidity burden (Charlson Index) in 28.2% of PLWH using EHR data, identifying key predictors such as age at diagnosis, tobacco use, low CD4+ and retention duration, achieving high predictive performance.
[54]	Yu et al. (2019)	Qualitative study	China	HIV and hepatitis C virus targets from therapeutic target and ChEMBL databases	HIV: 11 targets; Hepatitis C: 4 targets	Naïve Bayes, support vector machine	A multiple QSAR approach using naïve Bayes and SVM with MACCS and ECFP6 fingerprints predicted 20 multitarget HIV/HCV inhibitors with high accuracy (AUC 0.83–1.0); seven of nine novel compounds were experimentally validated to act on both HIV‐1 and HCV targets.
[55]	Zhang et al. (2018)	Retrospective study	Yale University	PLWH	1137	Ensemble learning, elastic net, LASSO, random forest, SVM, XGBoost	Ensemble ML identified 698 smoking‐associated CpGs predictive of HIV frailty (AUC = 0.73–0.78) and 5‐year mortality (HR = 1.46, *p* = 0.02) in PLWH, highlighting integrin signalling involvement.

Abbreviations: ADABoost, adaptive boosting; AI, artificial intelligence; ANFIS, adaptive neuro‐fuzzy inference system; ANN, artificial neural network; ARIMA, auto‐regressive integrated moving average model; ART, antiretroviral therapy; AUC, area under the curve; BRNN, bidirectional recurrent neural network; BSI, bloodstream infection; CASP, Critical Appraisal Skills Programme; CD4, cluster of differentiation 4; CHWs, community health workers; CNN, convolutional neural network; DT, decision tree; EFV, efavirenz; FBP, filtered back projection; GB, gradient boosting; GNB, Gaussian naïve Bayes; GOSDT, global optimal sparse decision trees; HCV, hepatitis C virus; HIV, human immunodeficiency virus; IDI, Infectious Disease Institute; KNN, k‐nearest neighbours; LASSO, least absolute shrinkage and selection operator; LMT, logistic model tree; LOCO, leave‐one‐covariate‐out inference; LR, logistic regression; ML, machine learning; MLP, multilayer perceptron; MLR, multiple linear regression; MSM, men who have sex with men; NLP, natural language processing; PLWH, people living with HIV; PNP, peripheral neuropathy; PRISMA, Preferred Reporting Items for Systematic Reviews and Meta‐Analyses; RDT, rapid diagnostic test; RF, random forest; RSN, resting state network; SHAP, Shapley additive explanations; SVM, support vector machine; TB, tuberculosis; TDM, therapeutic drug monitoring; TIV, trivalent inactivated influenza vaccine; VL, viral load; WTV, weighted total variation; XGB, extreme gradient boosting; YOLOX Nano, You Only Look Once Nano.

^a^ Unless otherwise stated, sample size values represent the number of participants; some entries specify number of tests, facilities or datasets.

The results are structured into four thematic areas: (1) HIV testing; (2) Retention in care and virological treatment response; (3) Clinical and immunological treatment outcomes; and (4) Testing and treatment support.

### 3.1 HIV testing

ML models have been employed to predict HIV and other sexually transmitted infection testing uptake [48], while predictive models have been applied to classify field‐based rapid diagnostic test images for HIV diagnosis [47]. The application of ML algorithms enhanced the specificity and positive predictive value in interpreting HIV rapid diagnostic tests, significantly reducing false positives compared to traditional visual methods [50]. AI‐assisted HIV self‐testing in Kenya demonstrated perfect sensitivity (100%) and high specificity of 98.8%, ensuring accurate interpretations and enhancing service delivery in pharmacy‐based HIV testing [56]. In rural South Africa, ML algorithms trained on 11,374 HIV rapid test images achieved 97.8% sensitivity and 100% specificity, outperforming human interpretation and supporting REASSURED mobile diagnostics [47].

### 3.2 Retention in care and virological treatment response

Predictive AI has been applied across multiple domains in HIV care, including retention in care [9, 15, 18, 22], viral load  suppression [16, 18, 20, 57] and virological failure [14, 21]. ML models have been employed to forecast missed ART appointments [34, 58] and predict clinic attendance [22], supporting adherence and retention strategies. ML models for retention in HIV care showed strong predictive performance across diverse contexts. Models achieved an area under the curve (AUC) of 0.69 for predicting clinic visit attendance in South Africa [59]; demonstrated the potential for identifying high‐risk patients with AUC values of 0.65 and 0.52, respectively, in Mozambique and Nigeria [9], and 75% accuracy [15]. A categorical boosting ML model, applied in a South African cohort, correctly identified 22,119 of 35,985 missed clinic visits, achieving a sensitivity of 62%, specificity of 67% and a positive predictive value of 20% [34, 58].

AI methods applied to predict VL suppression and identify patients in HIV care at risk of virological failure utilized algorithms such as RFs, LR and SVMs [16, 18, 20, 57]. These models, trained on routine patient data, including clinic attendance, viral load results and treatment history, generally achieved varying levels of accuracy (approximately 80−96%) and sensitivity (72−97%), but successfully identifying patients at a high risk for disengagement and highlighting the potential for targeted interventions to improve outcomes and optimize resource allocation. In South Africa, these models predicted retention and viral suppression with an AUC of up to 0.76 [59]. Models achieved 78% accuracy in identifying viral load hotspots in Kenya [16], 97% accuracy in predicting virological failure [20].

### 3.3 Clinical and immunological treatment outcomes

AI has shown promise in improving the monitoring of clinical outcomes in PLWH. Predictive models have been applied to predict clinical progression to AIDS [12], diagnose PLWH with laboratory‐confirmed bacteraemia or fungemia (hereafter, bloodstream infection [BSI]) [52], predict immune function recovery [35] and identify comorbidity predictors [53]. Additionally, various ML models have been employed to identify individuals at high risk for HIV‐associated morbidity—for example development of peripheral neuropathy [10] and mortality.

Models achieved 97% accuracy in predicting CD4 status in Ethiopia, and 90% accuracy in clustering regions by ART outcomes [20, 41]. In Yunnan, China, a study developed ML models to predict long‐term immune function changes in PLWH on ART. The SVM model performed best for CD4+ T cell recovery in patients with counts <200 cells/µl, while the RF model excelled for those with counts ≥200 cells/µl [35]. At Wenzhou Central Hospital in China, a study developed an AI diagnostic model to identify PLWH with BSI on the basis of eight clinical factors combined with age and gender. The model, built using the SVM with Radial Basis Function Kernel (svmRadial) algorithm, achieved an AUC curve of 0.916, sensitivity of 0.824 and specificity of 0.855, demonstrating excellent performance in diagnosing PLWH with BSI [52].

Furthermore, ML and AI have significantly enhanced diagnostic tools for HIV‐related conditions. In Malawi, a computer‐aided digital chest X‐ray system improved the timeliness of tuberculosis (TB) diagnoses, reducing the median time to TB treatment initiation from 11 days under standard care to just 1 day [25]. In South Africa, the CheXaid deep learning algorithm achieved 79% accuracy in diagnosing TB from chest X‐rays in PLWH, outperforming human clinicians (65% accuracy) and proving particularly valuable in resource‐limited settings [46].

In the United States, a study used deep neural networks to classify cognitive impairment and frailty in PLWH, achieving accuracies of 82–86% and 75%, respectively, while identifying critical cerebral blood flow patterns [38]. In a subsequent study, ML models were used to analyse resting state networks of the brain in 297 virologically suppressed PLWH and 1509 healthy controls, identifying that the salience and parietal memory networks effectively distinguished HIV status, while features of the frontal parietal network were associated with cognitive impairment, with minor variability in predictive strength observed across different age groups [37].

### 3.4 Testing and treatment support

AI chatbots have shown substantial potential in enhancing HIV services in Malaysia, Hong Kong and Singapore by providing accessible, stigma‐free and personalized support. In Malaysia, 93% of users found chatbots useful, and 79% expressed willingness to continue using them for HIV testing and related services [40]. A focus group with 31 MSM in Malaysia highlighted that chatbots addressing HIV testing were well‐received for their anonymity and ability to overcome systemic barriers like stigma and discrimination [23]. In Hong Kong, a trial involving 528 participants demonstrated that chatbots effectively promoted self‐testing uptake and provided counselling comparable to traditional methods [60]. In Singapore, chatbots offered accurate, comprehensive health information while reducing barriers to care, addressing stigma, and improving access to HIV prevention and treatment [61].

### 3.5 ML techniques

Various AI techniques have been extensively applied to enhance HIV care and research by addressing critical clinical and public health challenges (Table 2). Artificial neural networks (ANNs) [9, 12, 27, 29, 30, 35, 36, 37, 38] and RF [9, 10, 11, 12, 14, 16, 17, 18, 19, 20, 27, 29, 32, 33, 35, 39, 41, 51, 55] models have been utilized to predict treatment outcomes, viral load (VL) suppression and long‐term health trends. SVM [20, 22, 30, 33, 35, 41, 49, 51, 52, 54] and LR [9, 10, 11, 18, 22, 41, 48, 49, 51] have been applied to identify factors influencing clinic attendance, immune function and comorbidities. Deep learning models, including convolutional neural networks [25, 46, 50], assist in diagnosing co‐infections like TB through chest X‐rays, while bidirectional recurrent neural networks [21] capture temporal trends in VL and CD4 counts. Natural language processing [23, 26, 40, 60] has enabled the development of chatbots to promote HIV self‐testing and enhance patient engagement. Specialized techniques like YOLOX Nano [47] for interpreting HIV self‐test images, clustering methods for identifying socio‐behavioural predictors and gradient boosting [20, 44, 45] for predicting patient retention and ART interruptions, further highlight the capabilities of AI in HIV care. These applications showcase the versatility of AI in optimizing diagnosis, treatment and care delivery for PLWH.

**Table 2:** Machine learning techniques

Specific ML technique	Description	Applications	
Artificial neural networks (ANNs) [9, 12, 27, 29, 30, 35, 36, 37, 38]	Deep learning models inspired by biological neurons; excel in complex, non‐linear pattern recognition and are widely used in medical prediction tasks.	Predicting viral suppression and treatment outcomes [9, 12, 30]; predicting long‐term health outcomes [19, 27]; guiding resource allocation during high‐risk periods [36]; ART‐drug and demographic analysis [30]; predicting changes in immune function [35]; classifying cognitive impairment and frailty in PLWH [37, 38]; predicting and visualizing outcomes of HIV index testing [32]; predicting HIV/AIDS knowledge [29].	High predictive performance across varied HIV outcomes: accuracy up to 91.2%, AUC up to 0.86; widely applied.
Random forest (RF) [9, 10, 11, 12, 14, 16, 17, 18, 19, 20, 27, 29, 32, 33, 35, 39, 41, 51, 55]	Ensemble method using multiple decision trees; offers high accuracy, resistance to overfitting and handles missing data well.	Predicting viral suppression [17], CD4 count and treatment outcomes [9, 12, 14, 18, 20, 27, 41, 51]; stratifying patients for personalized care; predicting long‐term health outcomes [19]; trivalent inactivated influenza vaccine immunogenicity in children living with HIV using in vitro gene expression testing [33]; carotid artery plaques in PLWH [11]; changes in immune function [35]; predicting and visualizing outcomes of HIV index testing [32]; linking smoking‐related DNA changes to HIV outcomes [55]; predicting HIV/AIDS knowledge [29]; depressive symptoms [39]; peripheral neuropathy [10]; and clinic attendance [22].	Strong precision across tasks like viral load, immune function and clinic attendance; AUC up to 0.86; extensively used.
Support vector machines (SVMs) [20, 22, 30, 33, 35, 41, 49, 51, 52, 54]	Supervised ML model for classification and regression; effective in high‐dimensional spaces and binary outcomes.	Predicting viral suppression, CD4 count and treatment outcomes [20, 30, 35, 41, 49, 51]; trivalent inactivated influenza vaccine immunogenicity in children living with HIV using in vitro gene expression testing [33]; identified visual field defects by CD4 levels; linking smoking‐related DNA changes to HIV outcomes [55]; predicting BSI among PLWH [52]; multitarget inhibitors for HIV/HCV coinfection [54]; and clinic attendance [22].	Effective in classification and regression tasks with moderate to high accuracy in virologic and comorbidity predictions.
Logistic regression (LR) [9, 10, 11, 18, 22, 41, 48, 49, 51]	Interpretable model for binary classification; often used as a baseline for comparison.	Predicting viral suppression and treatment outcomes [9, 18, 31, 41, 49]; carotid artery plaques in PLWH [11]; HIV/AIDS knowledge [29]; peripheral neuropathy [10]; sexual behaviour [48]; and clinic attendance [22].	Widely adopted baseline; fair accuracy and interpretability but outperformed by non‐linear models in complex tasks.
Least absolute shrinkage and selection operator (LASSO regression) [14, 22, 53, 55, 62]	Regularization method for sparse models; performs variable selection and reduces overfitting.	Analysing risk factors for loss to follow‐up and mortality; predicting virological failure [14]; linking smoking‐related DNA changes to HIV outcomes [22, 55]; predicting comorbidity burden [53].	Useful in risk factor modelling; handles collinearity well; performance improves with large, structured datasets.
Elastic net regression [12, 22, 33]	Hybrid of ridge and LASSO regression; balances prediction accuracy and variable selection.	Analysing risk factors for loss to follow‐up and mortality [12]; predicting trivalent inactivated influenza vaccine (TIV) immunogenicity in children living with HIV using in vitro gene expression testing [33]; linked smoking‐related DNA changes to HIV outcomes [55]; predicting clinic attendance [22].	Combines LASSO and ridge strengths; reliable for variable selection; moderate performance in multi‐factor modelling.
Convolutional neural networks (CNNs) [25, 46, 50]	Specialized deep learning models for image data; state‐of‐the‐art in radiology and diagnostic imaging.	Assisting clinicians in screening and diagnosing TB using chest X‐rays in PLWH [25, 46]; improving accuracy in classifying HIV rapid test results [50].	High specificity in imaging tasks; effective in TB/HIV screening and test classification; state‐of‐the‐art in vision tasks.
Bidirectional recurrent neural networks (BRNNs) [21]	Neural networks that process sequential data forward and backward; ideal for time‐series clinical outcomes.	Capturing temporal trends in VL and CD4 counts; analysing sequential changes in patient health metrics; predicting HIV‐1 drug resistance [21].	Handles sequential data well; effective for resistance prediction; limited but strong use case for temporal data.
Gradient boosting (GB) [13, 34]	Ensemble method using decision trees trained sequentially; achieves strong performance in complex datasets.	Analysing sequential changes in patient health metrics; predicting interruptions in ART [34]; modelling survival rates and longitudinal health trends; identifying patterns in patient retention and mortality over time [20]; predicting weight change in ART‐experienced PLWH [13]; identifying neuroimaging, clinical and demographic features distinguishing frail from non‐frail PLWH [45]; predicting neurocognitive outcomes in children living with HIV [44].	Consistently high performance in ART interruption and patient outcome prediction; interpretable boosting mechanism.
Auto‐regressive integrated moving average (ARIMA) [36]	Time series forecasting model; effective for predicting incidence and trends in public health.	Predicting monthly HIV/AIDS incidence and guide resource allocation during high‐risk periods [36].	Good for time series forecasting; useful in incidence trend modelling; best in linear trends with stationary data.
Natural language processing (NLP) [23, 26, 40, 60]	AI technique for analysing and understanding human language; used in chatbots and patient interaction tools.	Assessing a chatbot's impact on HIV self‐testing uptake [23, 26, 60]; evaluating an AI chatbot's feasibility [40].	Effective for text and chatbot interventions; promising engagement tool in HIV prevention and testing campaigns.
Decision tree models [15, 19, 20, 27, 29, 41, 51]	Simple, interpretable models using rule‐based splits; helpful for initial exploratory analysis.	Exploring associations between demographic and clinical variables; providing interpretable insights for public health interventions [27]; predicting HIV outcomes [15, 20, 51]; HIV/AIDS knowledge [29]; and HIV outcomes [41].	Good interpretability; less complex, strong in demographic prediction; used for resource allocation insights.
Logistic model tree (LMT) [19]	Combines logistic regression with decision tree structure for interpretable classification.	Classifying anti‐HIV‐1 peptides based on their sequence‐related properties [19].	Integrates logistic regression with tree structure; useful for peptide classification; limited broader application.
Cox proportional hazards model (Cox regression) [25]	Statistical model for time‐to‐event outcomes; useful in retention, mortality and survival analysis.	Modelling survival rates and longitudinal health trends; identifying patterns in patient retention and mortality over time [25].	Best for survival analysis; used in longitudinal tracking; moderate complexity with interpretable outputs.
Bayesian modelling, expert system [27, 58]	Probabilistic models using prior knowledge and observed data; supports therapeutic drug monitoring.	Developing a computer‐based system to model and interpret plasma ART concentrations for therapeutic drug monitoring (TDM) [58]; predicting HIV outcomes [27].	Applied in specialized TDM models; effective in pharmacokinetics.
Linear regression (LR) [28]	Simple model for continuous outcomes; useful for associations but limited for non‐linear patterns.	Identifying correlations between immunologic signatures, clinical parameters and HIV DNA levels [28].	Simple correlations; foundational in multivariate studies; low performance in non‐linear, complex datasets.
Pairwise controlled manifold approximation projection (PaCMAP) [28]	Dimensionality reduction technique; useful for visualizing high‐dimensional clustering in immune data.	Reducing the dimensionality of immunophenotyping data to identify clusters of participants with distinct HIV reservoirs [28].	Dimensionality reduction strength; uncovers latent clusters; useful in HIV reservoir stratification.
Global optimal sparse decision trees (GOSDT) [28]	Sparse and interpretable decision trees optimized globally; supports rule‐based classification.	Predicting immunophenotypes by identifying sparse, interpretable decision rules linking immune and clinical features [28].	Sparse, interpretable rules; good for immunophenotype prediction; suited for clinical decision support.
Leave‐one‐covariate‐out inference (LOCO) [28]	Statistical method to evaluate the influence of each covariate on model output; supports model transparency.	Assessing the importance of individual covariates in predicting immunophenotypes and HIV DNA levels [28].	Feature importance analysis; enhances interpretability; complements other models in HIV DNA prediction.
Object detection (YOLOX Nano) [47]	Object detection model for interpreting images; used in HIV self‐testing result classification.	Interpreting HIV self‐testing images [47].	Reliable detection in image‐based testing; state‐of‐the‐art in self‐testing validation; limited generalizability.
Agglomerative hierarchical clustering [43]	Unsupervised clustering method; groups similar cases and visualizes socio‐behavioural HIV patterns.	Clustered countries by socio‐behavioural HIV predictors [43].	Cluster discovery from socio‐behavioural data; helpful in HIV policy insights; purely exploratory.
		

Abbreviations: ANNs, artificial neural networks; ARIMA, auto‐regressive integrated moving average; BRNN, bidirectional recurrent neural networks; CNNs, convolutional neural networks; GB, gradient boosting; GOSDT, global optimal sparse decision trees; LASSO, least absolute shrinkage and selection operator; LMT, logistic model tree; LOCO, leave‐one‐covariate‐out inference; LR, logistic regression; NLP, natural language processing; PaCMAP, pairwise controlled manifold approximation projection; RF, random forest; SVMs, support vector machines; YOLOX Nano, You Only Look Once, a deep learning object detection model.

## 4 DISCUSSION

The current review underscores the significant potential of predictive ML applications in advancing HIV care, aligning with broader analyses of ML in healthcare. Reviews, such as Obermeyer et al. on predictive analytics in general healthcare, and Topol on AI in clinical medicine, emphasize ML's versatility in improving patient outcomes through early intervention and resource optimization [63, 64]. Similarly, our analysis illustrates how ML models, using routine patient data, predict virological suppression, retention in care and clinical outcomes with moderate to high accuracy.

Previous reviews have noted ML's capacity to address critical healthcare gaps, such as predicting patients’ risk of disease progression, treatment interruption, adverse drug reactions, and loss to follow‐up and optimizing treatment pathways [65, 66, 67]. For example, Topol discusses ML's role in personalized medicine, which aligns with this review's findings on predicting individual patient outcomes, such as virological failure and immune function recovery [64]. The capacity of ML to address retention in care parallels findings in primary care reviews, where predictive models have improved appointment adherence and patient engagement strategies [63].

The use of AI in diagnostics, as highlighted in our analysis, mirrors broader findings in imaging and pathology. For example, the application of AI‐assisted diagnostic tools in TB and BSI detection aligns with reviews in oncology and radiology, which documented the enhancement of diagnostic accuracy and efficiency through deep learning models [68]. These advancements underscore ML's versatility in integrating clinical data and imaging for timely and precise diagnosis.

Previous reviews have identified challenges in the implementation of AI within clinical practice, including poor data quality, algorithmic bias and limited scalability of models across diverse settings [67, 69]. Issues such as the need for high‐quality training datasets, ethical considerations in model deployment and integration into clinical workflows have been extensively documented [64]. Furthermore, the introduction of AI into HIV care presents significant regulatory and ethical challenges, particularly concerning data privacy and potential biases in AI models [69, 70]. Ensuring patient confidentiality is paramount, as AI systems often require access to sensitive health information, raising concerns about data security and unauthorized use [71]. Moreover, AI models trained on non‐representative datasets may perpetuate existing health disparities, leading to biased outcomes in diagnosis and treatment [72].

To address the identified challenges, practical steps, including embedding equity into AI design from the outset by ensuring inclusive data, applying bias‐aware modelling strategies, auditing decision outcomes and institutionalizing fairness through policy, can help prevent the perpetuation of health inequities [73]. Moreover, clear regulatory frameworks and ethical guidelines should be developed to safeguard patient data privacy and support responsible AI deployment in HIV care [70, 74]. Strengthening collaborations between clinicians, data scientists and policymakers is essential for aligning AI innovations with frontline healthcare needs [63].

This synthesis reinforces the broader consensus that ML has the potential to transform healthcare delivery across various disciplines. While specific to HIV care, the findings reflect universal themes in ML applications, emphasizing the need for ongoing research and collaboration to maximize the benefits of these technologies in achieving better health outcomes globally.

AI and ML have the potential to revolutionize HIV care, particularly in sub‐Saharan Africa, where the epidemic remains most severe, and health systems face significant challenges. AI is transforming HIV care by enabling early identification of high‐risk patients, strengthening retention strategies, accelerating TB/HIV co‐infection diagnosis and expanding access to self‐testing, with the potential of driving better health outcomes in the most affected regions. As these technologies continue to evolve, their integration into healthcare systems could optimize resource allocation, enhance early intervention strategies and improve overall patient outcomes. However, to maximize their impact, AI‐driven solutions must be tailored to local contexts, address structural barriers and ensure equitable access, ultimately strengthening HIV care delivery in the regions that need it most.

Looking ahead, the next phase for AI in HIV care and research should focus on evaluating the long‐term impact of AI interventions on patient outcomes, health system efficiency and equity in HIV care, particularly in under‐resourced settings. Moreover, the development of locally relevant implementation frameworks grounded in ethical, regulatory and infrastructural realities will be essential to guide the responsible and sustainable integration of AI into HIV programmes.

The present review has several limitations. The heterogeneity of studies makes direct comparisons challenging due to variations in design, population and AI methods. Additionally, publication bias may influence findings, as negative results are less frequently reported. Only studies published in English were included due to resource constraints, which may have led to the exclusion of relevant studies published in other languages.

## 5 CONCLUSIONS

Our review highlights the transformative potential of ML applications in HIV treatment and care, demonstrating their effectiveness in predicting critical outcomes such as retention in care, virological treatment response and clinical complications. By leveraging routine patient data, ML models have proven valuable in optimizing resource allocation, improving diagnostic accuracy and enabling targeted interventions, particularly in resource‐limited settings. While significant advancements have been achieved, challenges such as data quality, algorithmic bias and integration into clinical workflows persist. Addressing these limitations through robust research, ethical considerations and scalable implementations will be crucial for maximizing the impact of ML on HIV care.

The findings of this work reinforce the broader potential of ML across healthcare disciplines, aligning with global efforts to enhance patient outcomes and achieve public health goals, including the UNAIDS 95‐95‐95 targets. Future research should focus on refining ML algorithms, fostering interdisciplinary collaborations and ensuring equitable access to AI‐driven innovations in HIV treatment and beyond.

## COMPETING INTERESTS

The authors declare that they have no competing interests related to this study.

## AUTHOR CONTRIBUTIONS

SN conceptualized the study, conducted the research, performed data extraction and analysis, wrote the manuscript and coordinated the overall study. EMM and ML reviewed the study, contributed to the study design, and assisted in defining the selection and exclusion criteria. JS reviewed the study with a specific focus on AI methods and contributed to refining the AI‐related methodology. LM designed the search strategy, conducted the literature search and reviewed relevant studies. TS conducted the quality appraisal of the selected studies, ensuring methodological rigour. TR reviewed the study, contributed to the study design, and played a key role in refining the selection and exclusion criteria.

## FUNDING

No funding was received for the conduct of this study or the preparation of this manuscript.

## DISCLAIMER

The views and opinions expressed in this study are those of the authors and do not necessarily reflect the official policy or position of any affiliated institution or organization.

## Supporting information




**File S1**: Search strategy

**File S2**: Quality appraisal

PRISMA 2020 Checklist

## Data Availability

All data used in this systematic review are derived from previously published studies, which are cited in the manuscript.

## Footnotes

* Numbers represent the count of included studies conducted in each country.
