# Supplementary material for: Artificial intelligence for HIV care: a global systematic review of current studies and emerging trends
Source: J Int AIDS Soc. 2025 Sep 24;28(10):e70045. doi: 10.1002/jia2.70045 (PMC12458397; doi:10.1002/jia2.70045)
Supplement: Supplementary file 1 — File S1: Search strategy [file JIA2-28-e70045-s002.docx]

**Search Strategy:**

**Document Types:** Article
**Language:** English
**Timespan:** 2014-2024

**PubMed:**
Search Query:
(("HIV care" OR "HIV treatment" OR "HIV management" OR "AIDS care" OR "AIDS treatment" OR "Antiretroviral therapy" OR "HIV/AIDS interventions" OR "HIV support" OR "HIV services") AND (y_10[Filter]))
AND
(("Artificial Intelligence" OR "AI applications" OR "Machine learning" OR "Automated systems" OR "Technology in healthcare" OR "Digital health" OR "Health informatics") AND (y_10[Filter]))
**Total:** 265

**Scopus:**
Search Query:
("HIV care" OR "HIV treatment" OR "HIV management" OR "AIDS care" OR "AIDS treatment" OR "Antiretroviral therapy" OR "HIV/AIDS interventions" OR "HIV healthcare" OR "HIV support" OR "HIV services")
AND
("Artificial Intelligence" OR "AI applications" OR "Machine learning" OR "Automated systems" OR "Technology in healthcare" OR "Digital health" OR "Health informatics")
**Total:** 355

**Web of Science:**
Search Query:
"HIV care" OR "HIV treatment" OR "HIV management" OR "AIDS care" OR "AIDS treatment" OR "Antiretroviral therapy" OR "HIV/AIDS interventions" OR "HIV healthcare" OR "HIV support" OR "HIV services"
AND
"Artificial Intelligence" OR "AI applications" OR "Machine learning" OR "Automated systems" OR "Technology in healthcare" OR "Digital health" OR "Health informatics"
**Total:** 314

**ProQuest Central:**
Search Query:
("HIV care" OR "HIV treatment" OR "HIV management" OR "AIDS care" OR "AIDS treatment" OR "Antiretroviral therapy" OR "HIV/AIDS interventions" OR "HIV healthcare" OR "HIV support" OR "HIV services")
AND
("Artificial Intelligence" OR "AI applications" OR "Machine learning" OR "Automated systems" OR "Technology in healthcare" OR "Digital health" OR "Health informatics")
**Total:** 2799
