# Supplementary material for: Artificial intelligence for HIV care: a global systematic review of current studies and emerging trends
Source: J Int AIDS Soc. 2025 Sep 24;28(10):e70045. doi: 10.1002/jia2.70045 (PMC12458397; doi:10.1002/jia2.70045)
Supplement: Supplementary file 2 — File S2: Quality appraisal [file JIA2-28-e70045-s003.docx]

# Table 1: CASP Checklist

| **Reference (Author, Year)** | **1** | **2** | **3** | **4** | **5** | **6** | **7** | **8** | **9** | **10** |
| --- | --- | --- | --- | --- | --- | --- | --- | --- | --- | --- |
| Aybar & Flores (2023) | Y | Y | Y | Y | Y | CT | CT | Y | Y | Y |
| Bala (2023) | Y | Y | Y | Y | Y | Y | Y | Y | Y | Y |
| Bisaso (2018) | Y | Y | Y | Y | Y | CT | Y | Y | Y | Y |
| Cheah (2024) | Y | Y | Y | Y | Y | Y | Y | Y | Y | Y |
| Chen (2023) | Y | Y | Y | Y | Y | Y | Y | Y | Y | Y |
| Chikusi (2022) | Y | Y | Y | Y | Y | CT | CT | Y | Y | Y |
| Cotugno (2020) | Y | Y | Y | Y | Y | Y | Y | Y | Y | Y |
| Domínguez-Rodríguez (2022) | Y | Y | Y | Y | Y | Y | Y | Y | Y | Y |
| Esber (2023) | Y | Y | Y | Y | Y | Y | Y | Y | Y | Y |
| Yu (2019) | Y | Y | Y | Y | Y | CT | CT | Y | Y | Y |
| Federico (2023) | Y | Y | Y | Y | Y | CT | CT | Y | Y | Y |
| Goicoechea et al. (2007) | Y | Y | Y | Y | Y | CT | CT | Y | Y | Y |
| Kozak (2007) | Y | Y | Y | Y | Y | Y | Y | Y | Y | Y |
| Li (2020) | Y | Y | Y | Y | Y | CT | CT | Y | Y | Y |
| Li et al. (n.d.) | Y | Y | Y | Y | Y | Y | Y | Y | Y | Y |
| Luckett (2019) | Y | Y | Y | Y | Y | Y | Y | Y | Y | Y |
| Luckett (2021) | Y | Y | Y | Y | Y | Y | Y | Y | Y | Y |
| Maskew et al. (n.d.) | Y | Y | Y | Y | Y | Y | Y | Y | Y | Y |
| MacPherson (2021) | Y | Y | Y | Y | Y | Y | Y | Y | Y | Y |
| Mamo (2023) | Y | Y | Y | Y | Y | Y | Y | Y | Y | Y |
| Marathe et al. (n.d.) | Y | Y | Y | Y | Y | Y | Y | Y | Y | Y |
| Roy et al. (n.d.) | Y | Y | Y | Y | Y | Y | Y | Y | Y | Y |
| Matta (2023) | Y | Y | Y | Y | Y | N | CT | Y | Y | Y |
| Koh (2024) | Y | Y | Y | Y | Y | N | N | Y | Y | Y |
| Mutai (2023) | Y | Y | Y | Y | Y | CT | CT | Y | Y | Y |
| Kagendi (n.d.) | Y | Y | Y | Y | Y | Y | Y | Y | Y | Y |
| Olatosi (2021) | Y | Y | Y | Y | Y | CT | CT | Y | Y | Y |
| Paul et al. (n.d.) | Y | Y | Y | Y | Y | Y | Y | Y | Y | Y |
| Peng (2022) | Y | Y | Y | Y | Y | Y | Y | Y | Y | Y |
| Poorinmohammad (2015) | Y | Y | Y | Y | Y | CT | CT | Y | Y | Y |
| Pranav (2020) | Y | Y | Y | Y | Y | Y | Y | Y | Y | Y |
| Rachel (2023) | Y | Y | Y | Y | Y | Y | Y | Y | Y | Y |
| Roche (2024) | Y | Y | Y | Y | Y | Y | Y | Y | Y | Y |
| Rodríguez-Aguilar et al. (n.d.) | Y | Y | Y | Y | Y | CT | CT | Y | Y | Y |
| Andresen et al. (n.d.) | Y | Y | Y | Y | Y | Y | Y | Y | Y | Y |
| Seboka (2023) | Y | Y | Y | Y | Y | Y | Y | Y | Y | Y |
| Semenova (2023) | Y | Y | Y | Y | Y | Y | Y | Y | Y | Y |
| Shi (2022) | Y | Y | Y | Y | Y | Y | Y | Y | Y | Y |
| Steiner (2020) | Y | Y | Y | Y | Y | CT | CT | Y | Y | Y |
| Stockman et al. (n.d.) | Y | Y | Y | Y | Y | CT | CT | Y | Y | Y |
| Tu et al. (n.d.) | Y | Y | Y | Y | Y | Y | Y | Y | Y | Y |
| Turbé (2021) | Y | Y | Y | Y | Y | CT | CT | Y | Y | Y |
| Wang et al. (n.d.) | Y | Y | Y | Y | Y | CT | CT | Y | Y | Y |
| Wang et al. (n.d.) | Y | Y | Y | Y | Y | Y | Y | Y | Y | Y |
| Mulyadi (n.d.) | Y | Y | Y | Y | Y | CT | CT | Y | Y | Y |
| Wu (2023) | Y | Y | Y | Y | Y | Y | Y | Y | Y | Y |
| Xianglong (2022) | Y | Y | Y | Y | Y | Y | Y | Y | Y | Y |
| Yang (n.d.) | Y | Y | Y | Y | Y | CT | CT | Y | Y | Y |
| Chen (2023) | Y | Y | Y | Y | Y | Y | Y | Y | Y | Y |
| Yu (2019) | Y | Y | Y | Y | Y | CT | CT | Y | Y | Y |
| Zhang (2018) | Y | Y | Y | Y | Y | Y | Y | Y | Y | Y |
| \| Legends \| \| \| --- \| --- \| \| Yes \| Y \| \| Can't Tell \| CT \| \| No \| N \| | | | | | | | | | | |
| \| **Questions** \| \| --- \| \| 1. Was there a clear statement of the aims of the research? \| \| 2. Is the methodology appropriate? \| \| 3. Was the research design appropriate to address the aims of the research? \| \| 4. Was the recruitment strategy appropriate to the aims of the research? \| \| 5. Was the data collected in a way that addressed the research issue? \| \| 6. Has the relationship between researcher and participants been adequately considered? \| \| 7. Have ethical issues been taken into consideration? \| \| 8. Was the data anyalysis rigourous enough? \| \| 9. Is there a clear statement of findings? \| \| 10. How valuable is the research? \| | | | | | | | | | | |
